# Typical Characterization of Commercial Camellia Oil Products Using Different Processing Techniques: Triacylglycerol Profile, Bioactive Compounds, Oxidative Stability, Antioxidant Activity and Volatile Compounds

**DOI:** 10.3390/foods11213489

**Published:** 2022-11-03

**Authors:** Jing Zeng, Weifei Wang, Ying Chen, Xuan Liu, Qingqing Xu, Suijian Qi, Dongming Lan, Yonghua Wang

**Affiliations:** 1 Department of Food Science and Engineering, School of Food Science and Engineering, South China University of Technology, Guangzhou 510640, China; 2 Sericultural and Agri-Food Research Institute, Guangdong Academy of Agricultural Sciences, Guangzhou 510610, China; 3 Guangdong Youmei Institute of Intelligent Bio-Manufacturing, Foshan 528226, China

**Keywords:** bioactive compounds, camellia oil, oil quality, oxidative stability, TAGs profile, volatile compounds

## Abstract

The processing technique is one of the key factors affecting the quality of camellia oil. In this study, camellia oils were obtained using four different processing techniques (cold-pressed, roast-pressed, fresh-pressed, and refined), and their triacylglycerols (TAGs) profile, bioactive compound (tocopherols, sterols, squalene, and polyphenols) level, oxidative stability, and volatile compounds were analyzed and compared. To further identify characteristic components in four camellia oil products, the TAG profile was analyzed using UPLC-QTOF-MS^E^. Five characteristic markers were identified, including OOO (*m/z* 902.8151), POL (*m/z* 874.7850), SOO (*m/z* 904.8296), PPL (*m/z* 848.7693), PPS (*m/z* 852.7987). Regarding the bioactive compound level and antioxidant capacity, the fresh-pressed technique provided higher α-tocopherols (143.15 mg/kg), β-sitosterol (93.20 mg/kg), squalene (102.08 mg/kg), and polyphenols (35.38 mg/kg) and showed stronger overall oxidation stability and antioxidant capacity. Moreover, a total of 65 volatile compounds were detected and identified in four camellia oil products, namely esters (23), aldehydes (19), acids (8), hydrocarbons (3), ketones (3), and others (9), among which pressed oil was dominated by aldehydes, acid, and esters, while refined oil had few aroma components. This study provided a comprehensive comparative perspective for revealing the significant influence of the processing technique on the camellia oil quality and its significance for producing camellia oil of high quality and with high nutritional value.

## 1. Introduction

Camellia oil is the edible oil extracted from the mature seeds of *Camellia oleifera* (*C. oleifera*), which has been widely cultivated for more than 2000 years in southern China [1]. As a woody oil with Chinese characteristics, it has the reputation of “Oriental Olive Oil” due to its extremely similar physicochemical properties and fatty acid composition to olive oil [2]. Generally, the quality of camellia oil plays a crucial role in consumer choices, which depends on their nutritional properties or organoleptic perspectives [3]. The nutritional value of camellia oil is related to its fatty acid composition and minor components, whereas the aroma is strongly influenced by volatile compounds [4]. Camellia oil contains abundant unsaturated fatty acids (>85%), especially oleic acid (65–84%), which plays a crucial role in reducing the incidence of cardiovascular diseases [5]. Moreover, camellia oil is viewed as a highly nutritional edible oil due to its various minor bioactive compounds, such as tocopherols, phytosterols, polyphenols, and squalene [6]. These minor bioactive compounds have been proven to have potential antimicrobial, antioxidant, anti-inflammatory, and anti-carcinogenic activities [7]. Flavor is an index to measure the quality of camellia oil [8]. The delicate and original flavor of camellia oil could improve the organoleptic perspectives so that it attracts numerous consumers [9]. In general, the levels of minor bioactive compounds and volatile compounds depend largely on processing techniques [10]. Hence, the retention of minor bioactive compounds and the types of volatile compounds have obtained increasing public concern.

Nowadays, different approaches have been proposed for the extraction of camellia oil, such as mechanical-pressed (cold-pressed, roast-pressed, fresh-pressed), commercially refined, aqueous enzymatic, and supercritical extraction [11]. Despite the success of the aqueous enzymatic and supercritical extraction in the laboratory, the mechanical pressing and commercial refinement are still the most widely used techniques for producing camellia oil in the industrial applications owing to their easy operation, high efficiency, and low cost [12,13]. Many studies have reported that processing techniques have a significant impact on the TAG profile, bioactive compounds, oxidative stability, and volatile compounds of edible oil. Sun et al. [14] found that hazelnut oils (*Corylus avellana* L.) extracted by different processing technologies (cold pressing, ultrasound-assisted hexane and enzyme-assisted aqueous solution) have significantly different TAGs (TAG 54:3, TAG 52:2, TAG 54:4, TAG 54:2, TAG 52:3, TAG 54:5) [14]. As far as the bioactive compounds are concerned, previous studies have shown that the tea seed oil (*Camellia sinensis* L.) obtained from cold pressing showed higher levels of total phenolics and α-tocopherol and exhibited stronger radical scavenging ability [12]. In contrast, the traditional refining processing significantly led to a series of losses (40–60%) of bioactive compounds in tea seed oil, which related to the high temperature in deodorization [15]. Flavor, as a crucial factor to attract consumers, exhibited a specific aroma under different processing techniques. For example, roasted and steamed camellia oil are mainly dominated by aldehydes and alcohols, while microwaved and roasted camellia oil are dominated by aldehydes and heterocyclic compounds [16]. Therefore, it is necessary to use appropriate processing techniques during oil extraction, because it could effectively prevent the loss of bioactive compounds and retain the unique and delicate flavor of oil.

Based on these considerations, this research aimed to investigate the effects of processing techniques (cold pressing, roast pressing, fresh pressing, and refinement) on the camellia oil quality. For this purpose, the TAGs profile, fatty acid composition, bioactive compounds, oxidation stability, free-radical scavenging capacity of camellia oil, and volatile compounds were quantified and compared. Meanwhile, qualitative and quantitative models were developed by UPLC-QTOF-MS^E^ and HS-SPME-GC-MS technology coupled with multivariate statistical analyses (PCA and cluster analysis) for fully understanding the differences in the camellia oil TAGs profile and volatile compounds among different processing techniques. This study will provide theoretical support to promote the development of the camellia oil industry, especially the market-set price and consumer choice of camellia oil. On the one hand, it provided an important reference for manufacturers to set reasonable prices for camellia oil according to their quality differences. Consumers may take the differences in the quality of camellia oil with different processing processes into consideration and then choose suitable oil to satisfy their needs.

## 2. Materials and Methods

### 2.1. Materials

Thirty-seven fatty acid methyl ester mixed standards, tocopherol standards (α-, δ-, and γ-tocopherol, purity > 95%), squalene standards (purity > 98%), β-sitosterol (purity > 98%), gallic acid, 2,2′-azino-bis (3-ethylbenzothiazo-line-6-sulfonic acid) diammonium salt (ABTS), and 2,2-diphenyl-1-pi-crylhydrazyl (DPPH) were provided by Shanghai ANPEL Laboratory Technologies Co., Ltd. (Shanghai, China). All standards were stored at −20 °C in the dark. All other chemicals and solvents were purchased from Sinopharm Chemical Reagent Co., Ltd. (Shanghai, China). Four camellia oils (cold-pressed, roast-pressed, fresh-pressed, and refined) were provided by Hunan Great Sanxiang Co., Ltd. (Hengyang, China), and the simplified processes (Figure S1) are as follows:

Cold-pressed: The camellia oils were obtained by pressing the dried camellia seeds at 60 °C.

Roast-pressed: The camellia oils were obtained by pressing the dried camellia seeds at 120–130 °C.

Fresh-pressed: The freshly picked camellia seeds were pressed immediately. Then, the camellia oils were obtained by centrifugation.

Refined: The extraction of camellia oil from camellia cake used petroleum ether (30–60 °C) as a solvent. Then, the solvents were removed in a vacuum and at a low temperature. Finally, the refined camellia oils were obtained by degumming (80 °C, 3% deionized water), deacidification (25% NaOH solution), decolorization (105 °C, 1.5% clay), deodorization (240 °C, 300 Pa, 2–3 h), and winterization (4 °C, 48 h).

### 2.2. TAGs Profile Analysis

Each oil sample (20 mg) was diluted to 1 μg/mL with isopropanol. The TAGs composition was identified and quantified by UPLC-Q-TOF-MS (Waters, Waters Xevo G2-XS QTOF, Milford, MA, USA) equipped with an Acuity UPLC CSH C18 column (100 mm × 2.1 mm × 1.7 μm; Waters, Milford, MA, USA). The analysis methods were modified according to Bang et al. [17]. Briefly, the HPLC conditions were as follows: the column temperature was maintained at 60 °C and the flow rate was 0.4 mL/min. Mobile phase A was a mixed solution of acetonitrile:water (60:40, *v*/*v*), and mobile phase B was a mixed solution of isopropanol:acetonitrile (90:10, *v*/*v*), both containing 0.1% formic acid and 10 mmol/L ammonium acetate. All reagents were of LC-MS grade. The gradient elution was as follows: the mobile phase B was 15% at 0 min; 0~2 min, 30% B; 2~2.5 min, 48% B; 2.5~11 min, 82% B; 11~11.5 min, 99% B;11~11.5 min, 99% B; 12~15 min, 15% B. The injection volume was 5 μL.

In the POS (+) mode of the electrospray ion source, the mass spectrometry analysis conditions were as follows: the capillary voltage was 3 kV, cone voltage was 40.0 V, and source offset was 80 V. Moreover, the source temperature was 120 °C, the desolvation temperature was 500 °C, and the desolvation gas was nitrogen at a flow rate of 800 L/h. The mass scan range was 50~1200 *m/z* (mass-to-charge ratio), and the acquisition mode was Q-TOF-MS^E^. The data were acquired by Waters Masslynx v4.2 software (Waters, Milford, MA, USA) and analyzed and processed by UNIF software (Waters, Milford, MA, USA) and Progenesis QI 3.0.3 (Waters, Milford, MA, USA).

### 2.3. Fatty Acid Analysis

The fatty acid composition of camellia oil was determined according to the previous study using an Agilent 7890A GC (Agilent, Santa Clara, CA, USA) equipped with an FID and CP Sil-88 column (60 m × 0.20 mm, 0.25 µm) [18]. Briefly, the methyl esterification of fatty acid was carried out by a transesterification reaction. Then, the supernatant was used for injection. The oven temperature was initially set at 180 °C for 5 min, then raised to 220 °C at 2 °C/min, subsequently increased to 220 °C at 5 °C/min and held for 10 min. The injection volume was 1 μL using 260 °C of injector temperature at a 1:30 split ratio. The carrier gas was high-purity nitrogen at 0.5 mL/min.

### 2.4. Bioactive Compounds Analysis

#### 2.4.1. Tocopherol, Sterols and Squalene Analysis

This method could be used for the simultaneous determination of tocopherols (α-, γ-, and δ-tocopherols), β-sitosterol and squalene in vegetable oils. The sample pretreatment method was modified based on the method of Yuan et al. [19]. In brief, samples (200 mg and containing 1% TBHQ) were weighed in tubes and mixed with 3 mL of 0.15 M NaOH-CH_3_OH solution. Then, the lower half of the tube was immersed for 40 min in a 70 °C bath and shaken every 5 min. After saponification, 2 mL of water and 3 mL of *n*-hexane (analytical grade) were added after cooling to room temperature. Subsequently, the aqueous phase was washed three times with *n*-hexane after being phase-separated in a separatory funnel. Finally, nitrogen blowing was used to remove *n*-hexane, reconstituted with methanol and isopropanol (*v:v* = 1:1, analytical grade), and filtered through a 0.22 μm nylon membrane filter.

The HPLC method was slightly modified from a previous method [20]. In this case, a Waters 2695 HPLC system (Waters Technologies, Milford, MA, USA) equipped with a UV detector and a reversed-phase C18 (250 mm × 4.6 mm× 5 µm) was used. The injection volume was 10 µL, and the mobile phase was 90% (A) MeOH and 10% (B) isopropanol at a flow rate of 1.0 mL min^−1^. All reagents of the mobile phase were of HPLC grade. The column was kept at a constant temperature (30 °C). Chromatograms were acquired at the absorbance maxima of tocopherols (296 nm) and phytosterols or squalene (205 nm). 

#### 2.4.2. Polyphenol Analysis

The extraction methods of polyphenols used the method of Wang et al. [21]. Briefly, 5.0 g oil was weighed into a centrifuge tube and then mixed with 5 mL *n*-hexane (analytical grade) and 5 mL methanol–water (60:40, *v*/*v*). Immediately, the mixture was shaken for 5 min in the dark using an HD-2500 multi-vortex mixer (Yooning, Hangzhou, China), and then the tube was centrifuged at 4000 rpm for 10 min at 4 °C. The methanol solution was separated, and it was described as the extraction solution. The operation was repeated three times. The analysis steps were as follows: 0.5 mL extracted solution mixed with 0.5 mL distilled water and 3.0 mL of 7.5% sodium carbonate solution. All solvents were of analytical grade. After 1 h of incubation at room temperature in the dark, the absorbance was measured at 765 nm. The results were expressed as mg of the gallic acid equivalent per kg of walnut oil sample (mg GAE/kg).

### 2.5. Oxidative Stability Analysis

Analysis of the oxidative stability was performed on Rancimat model 892 (Metrohm, Herisau, Switzerland). The induction periods (IPs) of the sample were assessed according to the previous study [22]. Camellia oil (3 g) was accurately weighed and placed inside the glass reaction vessel. The oil samples were roasted at 100 °C with a constant airflow of 10 L/h. 

### 2.6. Radical Scavenging Activity Analysis

The methods for determining the radical scavenging activity of the oil referred to the previous study [23]. The results of radical scavenging activity (DPPH, ABTS, FRAP) were expressed as Trolox equivalent antioxidant capacity (μmol TE/kg) using a Trolox calibration curve. The preparations of the extract were as follows:

Polar extract: 2.50 g oil was mixed with 4 mL methanol in a centrifuge tube, and then the mixture was shaken for 20 min (2000 rpm) by a DG-2500R multi-vortex mixer (Bajiu, Shanghai, China). The supernatant was separated and repeated the extraction three times. The supernatant was merged as the polar extract.

Non-polar extract: After polar extraction, the remaining part was retained as the non-polar extract.

Whole oil: Camellia oil (0.15 g) was accurately weighed and mixed with 5 mL ethyl acetate.

#### 2.6.1. DPPH Assay

Briefly, 2 mL of the polar extract was mixed with 2 mL of DPPH solution (40 mg/L in methanol). The mixture was placed in the dark at room temperature for 2 h, and then the DPPH absorbance was measured at 517 nm. The analysis of non-polar extract and whole oil was similar to that of the polar extract, and ethyl acetate was used as a solvent instead of methanol.

#### 2.6.2. ABTS Assay

The ABTS reagent was prepared as follows: 25 mL 7 mmol/L ABTS was mixed with 440 μL 2.45 mmol/L potassium persulfate solution and placed in the dark for 12–16 h at 25 °C. Then, the mixture solution was diluted to an absorption of 0.700 ± 0.020 at 734 nm by methanol. A 200 μL polar extract was mixed with 4 mL ABTS solution in the dark for 20 min at 25 °C. The absorbance was measured at 734 nm.

#### 2.6.3. FRAP Assay

The FRAP reagent preparation was as follows: 10 mmol/L TPTZ solution, 20 mmol/L FeCl_3_·6H_2_O solution, and 0.1 mol/L sodium acetate buffer solution (pH 3.6) were mixed at a 1:10:1 (*v*/*v*/*v*) ratio. A 300 μL oil polar extract was added to 2 mL of FRAP reagent and fixed to 10 mL with distilled water. After that, the mixture was heated at 37 °C for 10 min. The absorbance was measured at 593 nm.

### 2.7. Volatile Compounds Analysis

The volatile compounds of camellia oils were extracted using the headspace solid phase microextraction (HS-SPME) described by Liu et al. [9]. The oil sample (3.00 g) and 1,2-dichlorobenzene (132.64 μg/mL) were mixed in a 15 mL headspace sampling bottle and kept at 60 °C (30 min). The volatile aroma compounds present in the headspace of the vial were extracted by SPME using a 1 cm, 85 μm carboxen/polydimethylsiloxane (CAR/PDMS) fiber (Supelco, Inc., Bellefonte, PA, USA) for 30 min at 60 °C. 

The volatile compounds were analyzed using a GC-MS (TQ8050, Shimadzu, Kyoto, Japan) equipped with an SH-Rxi-5MS column (30 m × 250 μm × 0.25 μm, Shimadzu, Japan). The fiber was desorbed into the GC injection port for 5 min at 250 °C and analyzed by GC−MS using the splitless injection mode. The carrier gas was helium (99.999%) with a flow rate of 1.0 mL/min. The operating conditions were as follows: it kept at 35 °C for 2 min, and then heated to 120 °C (held for 2 min) at a rate of 8 °C/min; it was subsequently heated to 150 °C (held for 2min) at a rate of 5 °C/min; finally, it was further increased to 230 °C (held for 7 min) at a rate of 10 °C/min. The GC−MS transfer line and the ion source were set at 250 °C. Data acquisition was performed in the full-scan mode (scan range of *m/z* 50−450. All compounds were identified according to the NIST 2017 mass spectral library and authentic standards.

### 2.8. Statistical Analysis

The data were reported as the mean value ± standard deviation (SD) from three replicates of each sample. Meanwhile, all results were compared by one-way analysis of variance (ANOVA) using IBM SPSS Statistics 24.0. Correlations between different variables were calculated using a two-tailed Pearson bivariate correlation with significance levels of 0.01 (**) and 0.05 (*). Progenesis QI 3.0.3 (Waters) software was used to perform peak detection, screening, alignment, noise filtering, and other preprocessing on the original data to obtain a two-dimensional peak original data matrix. Then, it was used to normalize the data, followed by principal component analysis (PCA) unsupervised pattern recognition, projections to latent structures discriminant analysis (PLS-DA) supervised pattern analysis, and permutation tests to check whether the model was overfitted.

## 3. Results and Discussion

### 3.1. TAGs Profile Differences in Different Processing Techniques of Camellia Oil

#### 3.1.1. TAGs Profile Analysis

As shown in Table 1, the TAGs profile of camellia oil was identified and quantified by UPLC-Q-TOF-MS^E^. As a characteristic TAGs composition of camellia oil, the OOO (40.51–46.97%) almost accounted for almost half of the TAGs content, which was in accordance with the results reported by Wei et al. [24]. Obviously, the major TAGs composition also included POO (17.81–19.18%), followed by SOO (5.27–6.24%), OOL (5.96–7.16%), PPO (3.81–4.14%), OOLn (3.00–3.35%), PSO (2.41–2.68%), and SSO (1.34–1.92%). In addition, other minor TAGs were identified and quantified, including SSL, SSO, LLLn, LLnLn, PSS, PPLn, PLLn, and PLnLn (less than 1%). Significant differences in the TAGs profile were observed in oil samples extracted by different processing techniques (*p* < 0.05). In detail, camellia oil obtained from fresh pressing showed the highest levels of OOO (46.97 ± 1.02%) and the lowest OOL, SOO, and POL, which accounted for 6.01 ± 0.26%, 5.30 ± 0.03%, and 2.86 ± 0.11% of the total TAGs, respectively. In particular, it was observed that pressed camellia oil exhibited unique molecular TAGs (LLLn, LLnLn, and PPLn), while refined camellia oil showed distinctive PLLn and PLnLn. These characteristic TAGs with low content have the potential to distinguish between different processing techniques of camellia oils. The differences in TAGs could be attributed to the fact that the high temperature of refining promotes the acyl migration. It has been reported that the degree of acyl transfer increased from 85.5% to 98.57% when the reaction temperature increased from 50 °C to 80 °C [25].

The influence of processing techniques on the fatty acid composition (%) in camellia oil samples is shown in the Supplementary Material (Table S1). The main fatty acid composition of oil samples was oleic acid (79.50–79.97%), followed by linoleic acid (8.32–8.99%), palmitic acid (8.11–8.50%), stearic acid (2.07–2.23%). In addition, four fatty acids, namely linolenic acid, behenic acid, cis-11-eicosenoicacid, and arachidic acid, were found in relatively low amounts (<1%) in the oil samples. However, no significant difference was observed in fatty acids among the four processing techniques, which was in accordance with the previous report [26]. It is worth noting that trans fatty acid (TFA) was revealed in oil samples from four processing techniques, where the refined camellia oil showed the highest TFA content (0.17%). The formation of TFA may be related to the double-bond trans-isomerization owing to the high temperature during the refining process [27].

In this study, camellia oils were defined as edible oil with high-level unsaturated fatty acids (88.79–89.19%) and low-level saturated ones (9.00–9.35%) according to the TAGs profile and fatty acid composition. Generally, the oxidative stability of camellia oil is positively correlated with the number of double bonds. As characteristic fatty acid of camellia oil, oleic acid and linoleic acid could increase the risk of oxidation since they contain at least one double bond. Furthermore, the oxidative stability of camellia oil was also closely related to the species and content of bioactive compounds according to the reported research [28]. Therefore, it is necessary to investigate the retention of bioactive compounds in camellia oil from different processing techniques.

#### 3.1.2. Multivariate Statistical Analysis

Multivariate statistical analyses, including principal component analysis (PCA) and partial least-squares-discrimination analysis (PLS-DA), are powerful tools that are widely used to study the statistical regularity of interdependence among multiple variables [29]. The PCA could reduce the complexity of the data and identify the most important features, while PLS-DA models are extremely useful for Q-makers discovery and models interpretation [30]. The PCA and PLS-DA models can also be used for adulteration identification of edible oils based on comprehensive analysis of the TAGs profile [31]. Despite five characteristic TAGs molecules initially being analyzed, the entire TAGs profile still requires further analysis to screen for markers contributing to the discrimination between camellia oils obtained by different processing techniques.

**Figure 1:**
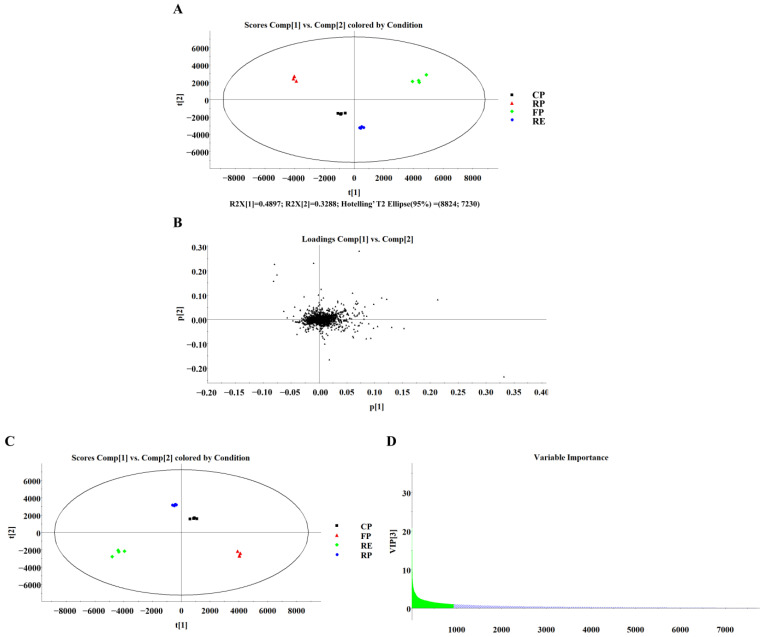
PCA score plot (**A**); PCA loading plot (**B**); PLS-DA scores plot (**C**); PLS-DA variable importance plot (**D**). CP, cold-pressed; RP, roast-pressed; HP, fresh-pressed; RE, refined.

In this study, the PCA model in unsupervised mode was used to explore the differences between group and group trends. The PCA score plot was shown in Figure 1A; no outliers were observed within Hotelling’s T2 (T2 critical 95%), illustrating that the data were reliable. A total of three principal components were analyzed, explaining a total of 88% of total variances, R2X (cum). The difference between treatment groups is related to the distance between samples. Being clustered together means higher similarity, while being scattered means greater differences. It was observed that the camellia oils from different processing techniques were completely distinguished. 

To further reinforce differences between the groups, the supervised PLS-DA was performed. The PLS-DA model exhibited excellent fit and good predictive capability, with R2 = 99% and Q2 = 98%, respectively. The PLS-DA loading is shown in Figure 1B, and the variables farther from the center origin had a greater impact on the difference between groups. The PLS-DA score plot (Figure 1C) was able to effectively distinguish clear differences among the four groups. Furthermore, the preliminary screening of compounds with a large contribution to the separation between groups was based on variable importance in projection (VIP > 1, green part), as shown in Figure 1D. Meanwhile, the screening of the potential differential marker compounds was also combined with *p* < 0.05 (mathematical statistical significance). As shown in Table 2, five variables were screened based on VIP > 1 and *p* < 0.05, including OOO (*m/z* 902.8151), POL (*m/z* 874.7850), SOO (*m/z* 904.8296), PPL (*m/z* 848.7693), and PPS (*m/z* 852.7987). The mass error of each compound was less than 5 ppm. It was remarkable that these significantly different TAGs could be used as potential markers to further understand the differences in cold-pressed, roast-pressed, fresh-pressed, and refined camellia oil.

### 3.2. Bioactive Compounds Differences in Different Processing Techniques of Camellia Oil

#### 3.2.1. Tocopherol

Tocopherols are important potential antioxidants during the peroxidation of unsaturated lipids [32]. As shown in Table 3, the content of α-tocopherol ranged from 3.27 to 143.15 mg/kg, with the processing techniques found to be ranked in the order: fresh-pressed > cold-pressed > roast-pressed > refined. Regarding δ-tocopherol and γ-tocopherol, both were not found in four camellia oil samples, indicating that α-tocopherol is the main form present in camellia oil [33]. The α-tocopherol has the highest in vivo antioxidant activity in four tocopherol isomers, playing an important role against lipid peroxidation. However, the α-tocopherol showed the lowest stability in four isomers, reaching a percentage of degradation of 33.77% in 48 h of 180 °C heating in rice bran oil [34]. The processing conditions may explain for such a huge difference in tocopherol. The α-tocopherol degraded rapidly when the deodorization temperature reached over 200 °C during the refining process of camellia oil [15].

#### 3.2.2. Polyphenols

The polyphenols can be used as an essential reference to evaluate the quality of camellia oil due to its antioxidant properties [35]. As shown in Table 3, significant differences (*p* < 0.05) were observed in the content of polyphenols detected in different processing techniques. Specifically, the polyphenols content of pressed camellia oils ranged from 19.15 to 35.38 mg/kg, while that of refined oil was not detected. The results indicated that processing techniques significantly affected the retention of polyphenols. On the one hand, the polyphenols dropped dramatically or were eliminated entirely in the high-temperature deodorization of the refining process due to their poor thermal stability, with an approximately 30% reduction when exposed to high temperatures for 4 h [36]. On the other hand, polyphenols could not be retained excellently in the oil phase because of their hydrophilic properties during the oil extraction process, and most of them remained in the camellia cake (around 1.5%) after mechanical pressing [37].

#### 3.2.3. Squalene

Squalene, a natural lipid belonging to the terpenoid family, was first found in the liver of marine sharks, mainly in marine animals or plants and their products [38]. The squalene content obtained in camellia oils with different processing techniques ranged from 25.21 to 106.01 mg/kg (Table 3), ranked in the following order: cold-pressed > fresh-pressed > roast-pressed > refined. The remarkable differences might be due to the fact that the content of antioxidant substances in oils depends not only on the cultivar and state of ripeness but also on the extraction and refining processes utilized [39]. The results showed that fresh-pressed technology was more favorable for the preservation of squalene.

#### 3.2.4. β-Sitosterol

Phytosterols are natural endogenous antioxidants, which have good oxidation and thermal stability and can effectively inhibit oil rancidity [40]. It is reported that the phytosterol content in the diet could lower blood cholesterol and triglycerides levels, which play an essential role in preventing cardiovascular diseases [41]. β-sitosterol is the most abundant phytosterol present in edible oil. Table 3 shows the retention of β-sitosterol under different processing techniques. Among the four processing techniques, fresh pressing and cold pressing showed superior retention of β-sitosterol, reaching up to 93.20 and 92.75 mg/kg, respectively. Roast-pressed oil showed a greater loss of β-sitosterol when compared with fresh-pressed and cold-pressed techniques, displaying 33.2 mg/kg. It is worth noting that β-sitosterol was not detectable in refined camellia oil. The adsorption of sterols during bleaching and the removal of sterols during the vacuum distillation step of deodorization are considered the main reasons for the losses of β-sitosterol during the refining process [42]. The results suggested that fresh-pressed and cold-pressed technologies are more favorable for the retention of β-sitosterol.

#### 3.2.5. Oxidative Stability and Radical Scavenging Activity Analysis

**Figure 2:**
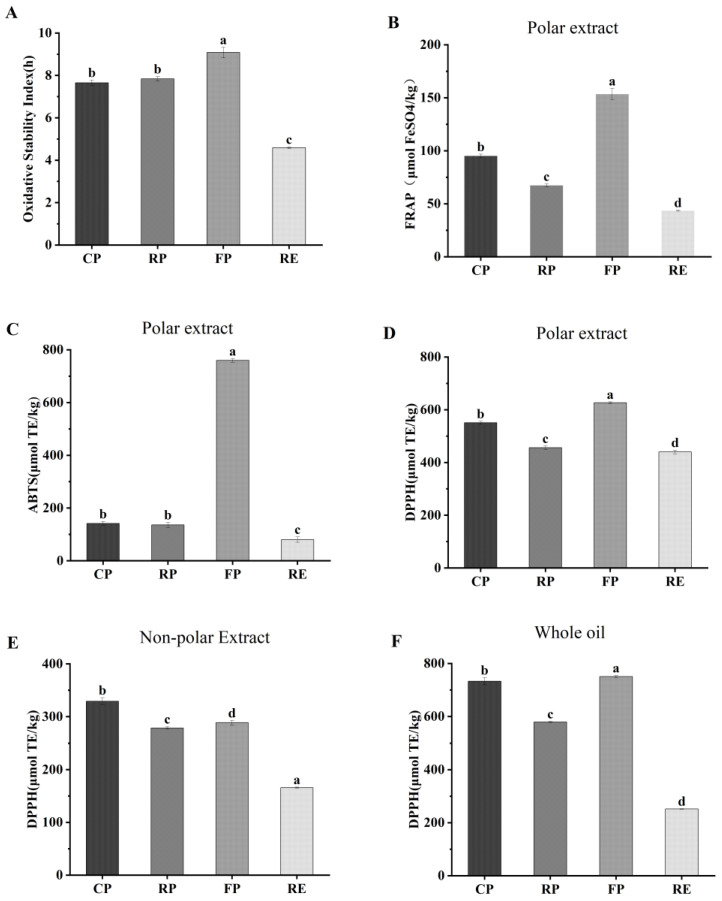
Free-radical scavenging capacity of camellia oil. (**A**) Oxidative stability index (OSI) of camellia oil; (**B**) FRAP assays of polar components in camellia oil; (**C**) ABTS assays of polar components in camellia oil; (**D**) DPPH assays of polar components in camellia oil; (**E**) DPPH assays of non-polar components in camellia oil; (**F**) DPPH assays of whole camellia oil. The superscript letters indicate the statistical difference in rows at a significant level of 5%. CP, cold-pressed camellia oil; RP, roast-pressed camellia oi; HP, fresh-pressed camellia oil; RE, refined camellia oil.

The oxidative stability and free-radical scavenging capacity of camellia oil are shown in Figure 2. The results indicated that fresh-pressed oil (9.08 h) had the highest oxidative stability, while the lowest oxidative stability was observed in refined oil (4.59 h) (Figure 2A). Interestingly, the oxidative stability of roast-pressed camellia oil (7.84 h) was slightly better than that of cold-pressed oil (7.65 h), which was due to the fact that the initial acid value of roast-pressed oil was lower than that of cold-pressed oil. Coincidentally, similar results were presented in flaxseed oil reported by Zhang [7], in which the oxidative stability index of roast-pressed oil (13.9 h) was higher than that of cold-pressed oil (10.1 h) during accelerated oxidation processing.

The free-radical scavenging capacity of the different oils was assessed by FRAP, ABTS, and DPPH assays, as shown in Figure 2B–D, respectively. The polar extract of fresh-pressed oil expressed the highest free-radical scavenging capacity in terms of DPPH and ABTS assays (626.6 and 759.7 μmol TE/kg), followed by cold-pressed and roast-pressed oil, whereas the polar extract of refined oil showed the lowest DPPH and FRAP free-radical scavenging capacities (440.06 and 80.31 μmol TE/kg). Moreover, the non-polar extract of cold-pressed oil and the whole oil of fresh-pressed both were the highest according to DPPH assay (329.15 and 750.69 μmol TE/kg, respectively). The results indicated that fresh-pressed camellia oil had the highest antioxidant capacity according to the comprehensive oxidative stability and free-radical scavenging capacity for retaining bioactive compounds effectively. However, the antioxidant capacity of refined camellia oil was not good when compared with other processing techniques. Similar research has reported that the radical scavenging capability of the polar extracts in refined camellia oil was lower than that of the polar extracts in virgin camellia oil according to the FRAP assay results [43].

The correlations between bioactive compounds, oxidative stability, and antioxidant activity are presented in Table 4. The oxidative stability index (OSI) showed a positive correlation with the level of α-tocopherol, squalene, β-sitosterol, and polyphenols. It is worth noting that all bioactive compounds had a strong positive correlation with the free-radical scavenging capacity, such as α-tocopherol exposing a significant positive correlation with DPPH (polar extract r = 0.977, non-polar extract r = 0.754 and whole oil r = 0.907), FRAP (r = 0.972), ABTS (r = 0.821) at the 0.01 level. In addition, a positive correlation was also found between bioactive compounds and FRAP as well as ABTS. Most bioactive compounds were recognized as extremely critical antioxidants in edible oil, such as α-tocopherol. Therefore, the maximum retention of bioactive compounds was beneficial for the oxidative stability of the oil during storage.

### 3.3. Volatile Compounds Differences in Different Processing Techniques of Camellia Oil

**Figure 3:**
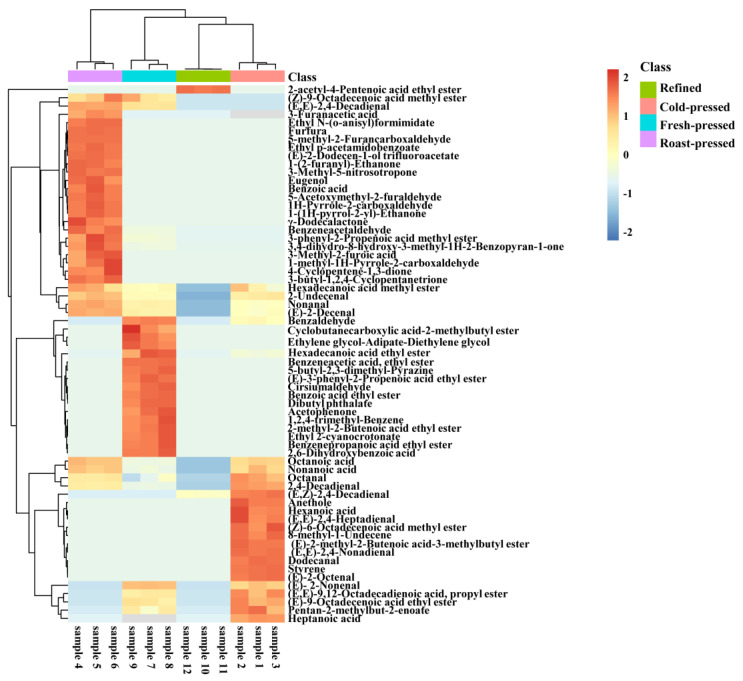
Cluster heat map of volatile compounds of camellia oil by different processing techniques.

The cluster heat map further revealed the differences among camellia samples with different processing techniques based on the content of 65 volatile compounds, as shown in Figure 3. The cluster heat map reflected the trend of changes in the concentration of volatile compounds in camellia oil samples from different processing techniques. A redder color represented a higher concentration of this volatile compound in the group, while a darker blue represented a lower level of this volatile compound in the group. The volatile components corresponded to each row, and camellia oil samples with different processing techniques corresponded to each column. It is worth noting that significant differences in the aroma compounds present in camellia oil using different processing techniques were observed. The camellia oil samples with different processing techniques were divided into 4 categories: cold-pressed, roast-pressed, fresh-pressed, and refined, and they could be distinguished by the color of the cluster map based on volatile compounds.

**Figure 4:**
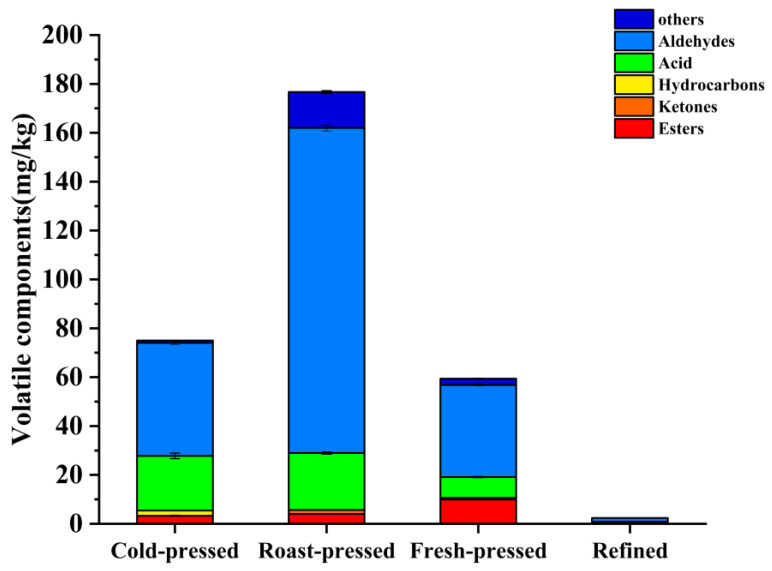
Classification of volatile compounds in camellia oil with different processing techniques.

To further analyze the differences in volatile compounds in four camellia oil products, we divided the volatile compounds of four camellia oil products into six categories according to their properties. The categories of volatile components of camellia oil by different processing techniques are shown in Figure 4. A total of 65 volatile compounds were detected and identified, including 23 esters, 19 aldehydes, 8 acids, 3 hydrocarbons, 3 ketones, and 9 others (Table S2). Specifically, roast-pressed camellia oil showed the highest levels of volatile compounds, while refined camellia oil had few volatile compounds. In pressed samples, cold-pressed oil was dominated by aldehydes (46.46 mg/kg) and acid (22.37 mg/kg), and roast-pressed oil was mainly dominated by aldehydes (132.8 mg/kg), acid (23.30 mg/kg) and esters (4.02 mg/kg), while fresh-pressed camellia oil had aldehydes (37.43 mg/kg) and esters (9.95 mg/kg) as the dominant volatile compounds.

In detail, there were 11 main aroma components in cold-pressed camellia oil, including 2-Undecenal (11.47 mg/kg), Octanoic acid (10.08 mg/kg), 2,4-Decadienal (10.03 mg/kg), Nonanoic acid (9.79 mg/kg), (E)-2-Decenal (7.55 mg/kg), Nonanal (6.43 mg/kg), (E,Z)-2,4-Decadienal (5.59 mg/kg), (E,E)-2,4-Heptadienal (2.33 mg/kg), Benzaldehyde (2.16 mg/kg), (E)-9-Octadecenoic acid ethyl (1.78 mg/kg), and Styrene (1.71 mg/kg), which were described as “grassy”, “fatty”, and “green”. Meanwhile, there were eight main aroma components in roast-pressed camellia oil, namely Furfural (62.61 mg/kg), 2-Undecenal (14.4 mg/kg), (E)-2-Decenal (13.40 mg/kg), 5-methyl-2-Furancarboxaldehyde (13.32 mg/kg), Octanoic acid (10.95 mg/kg), Nonanoic acid (10.18 mg/kg), 2,4-Decadienal (6.46 mg/kg), and Benzeneacetaldehyde (3.75 mg/kg), respectively. Furfural was only detected in the roast-pressed oil, and its aroma is described as “sweet popcorn” and “wood”. In addition, the main aroma of fresh-pressed camellia oil included 2-Undecenal (9.45 mg/kg), (E)-2-Decenal (9.09 mg/kg), Nonanal (7.86 mg/kg), Octanoic acid (4.30 mg/kg), Nonanoic acid (4.13 mg/kg), 2,4-Decadienal (2.93 mg/kg), Benzoic acid ethyl ester (2.90 mg/kg), (E, E)-2,4-Decadienal (1.50 mg/kg), and 2-methyl-2-Butenoic acid ethyl ester (1.08 mg/kg). The aromas of fresh-pressed oil samples were described as “fat”, “green”, and “vanilla”, which is the combination of the typical oil aroma and the natural fragrance of Camellia oleifera. Regarding refined camellia oil, only five components were detected, namely Nonanal (0.13 mg/kg), (E)-2-Decenal (0.32 mg/kg), (E, Z)-2,4-Decadienal (0.75 mg/kg), 2-Undecenal (0.33 mg/kg) and Cirsiumaldehyde (0.77 mg/kg), and these volatile components have no significant influence on the overall aroma of camellia oil. In brief, the pressed processing retained the original fragrance of camellia seed, while the refining processing resulted in a significant loss of flavor. These results demonstrated that the processing techniques had an important influence on the camellia aroma compound, and volatile compounds decreased rapidly after refining processing.

## 4. Conclusions

This study exhibited the significant impacts of different processing techniques (cold-pressed, roast-pressed, fresh-pressed, and refined) on the quality of camellia oil. Firstly, five TAGs molecules were screened using UPLC-QTOF-MS^E^ technology, including OOO (*m/z* 902.8151), POL (*m/z* 874.7850), SOO (*m/z* 904.8296), PPL (*m/z* 848.7693), PPS (*m/z* 852.7987). Secondly, the fresh-pressed camellia oil showed a higher level of retention of bioactive compounds (tocopherol, phytosterols, squalene, and polyphenols), which also exhibited better oxidative stability and free-radical scavenging capacity. Finally, volatile compounds in camellia oil by different processing techniques were identified by HS-SPME-GC-MS, and it was found that cold-pressed and roast-pressed camellia oil were dominated by aldehydes and acid, and fresh-pressed camellia oil had aldehydes and esters as the dominant volatile compounds, while refined camellia oil had few aroma components. Overall, this study provided insight into the understanding of the effect of different processing techniques on camellia oil quality.

## Figures and Tables

**Table 1:** TAGs profile of camellia oils (%).

TAG Profile	Cold-Pressed	Roast-Pressed	Fresh-Pressed	Refined
SSL	0.04 ± 0.00 ^c^	0.04 ± 0.00 ^c^	0.05 ± 0.00 ^b^	0.08 ± 0.00 ^a^
SSS	0.57 ± 0.05 ^a^	0.49 ± 0.04 ^b^	0.55 ± 0.02 ^a^	0.29 ± 0.02 ^c^
SSO	1.92 ± 0.08 ^a^	1.73 ± 0.13 ^b^	1.34 ± 0.10 ^c^	0.46 ± 0.00 ^d^
SOO	6.40 ± 0.30 ^b^	6.89 ± 0.24 ^a^	5.30 ± 0.03 ^c^	6.27 ± 0.28 ^b^
OOO	45.43 ± 1.07 ^ab^	44.38 ± 0.55 ^b^	46.97 ± 1.02 ^a^	40.51 ± 1.27 ^c^
OOL	6.62 ± 0.10 ^c^	7.16±0.06 ^b^	6.01± 0.26 ^d^	8.74 ± 0.26 ^a^
OOLn	3.09 ± 0.10 ^b^	3.35 ± 0.03 ^a^	3.00 ± 0.14 ^b^	2.42 ± 0.13 ^c^
OLLn	1.85 ± 0.05 ^a^	1.90 ± 0.02 ^a^	1.85 ± 0.09 ^a^	1.33 ± 0.08 ^b^
LLLn	0.49 ± 0.01 ^a^	0.28 ± 0.24 ^b^	0.48 ± 0.03 ^a^	ND
LLnLn	0.09 ± 0.01 ^a^	0.18 ± 0.19 ^a^	0.10 ± 0.02 ^a^	ND
PSS	1.07 ± 0.07 ^a^	1.01 ± 0.06 ^a^	0.88 ± 0.04 ^ab^	0.67 ± 0.06 ^ab^
PSO	2.68 ± 0.26 ^a^	2.61 ± 0.07 ^a^	2.41 ± 0.13 ^a^	2.43 ± 0.12 ^a^
POO	17.81 ± 0.36 ^c^	18.14 ± 0.21 ^b^	18.18 ± 0.10 ^b^	19.42 ± 0.63 ^a^
POL	3.19 ± 0.07 ^a^	3.24 ± 0.05 ^a^	2.86 ± 0.11 ^b^	3.44 ± 2.38 ^a^
POLn	1.54 ± 0.04 ^a^	1.63 ± 0.04 ^a^	1.69 ± 0.05 ^a^	1.59 ± 0.06 ^a^
PPS	0.72 ± 0.01 ^a^	0.77 ± 0.02 ^a^	0.81 ± 0.08 ^a^	0.73 ± 0.04 ^a^
PPO	4.05 ± 0.03 ^b^	3.81 ± 0.09 ^b^	3.97 ± 0.17 ^b^	7.44 ± 0.21 ^a^
PPL	1.17 ± 0.06 ^b^	1.12 ± 0.05 ^b^	1.20 ± 0.02 ^b^	2.28 ± 0.08 ^a^
PPLn	0.20 ± 0.00 ^a^	0.18 ± 0.00 ^a^	0.02 ± 0.02 ^b^	ND
PPP	1.09 ± 0.02 ^b^	1.09 ± 0.03 ^b^	1.42 ± 0.05 ^a^	1.42 ± 0.00 ^a^
PLLn	ND	ND	ND	0.24 ± 0.19 ^a^
PLnLn	ND	ND	ND	0.26 ± 0.19 ^a^

The superscript letters indicate the statistical difference in rows in significant level at 5%. ND, not detected. P, palmitic; S, stearic; O, oleic; L, linoleic; Ln, linolenic.

**Table 2:** Markers identified in different processing methods of camellia oils.

Formula	Description	*m/z*	Mass Error (ppm)	Adducts	Anova (p)	q Value	VIP
C_57_H_104_O_6_	OOO	902.8151	−2.2338	[M+NH_4_]^+^	6.19 × 10^−10^	1.01 × 10^−10^	13.0598
C_55_H_100_O_6_	POL	874.7850	−0.9318	[M+NH_4_]^+^	5.83 × 10^−4^	4.61 × 10^−5^	6.9993
C_57_H_106_O_6_	SOO	904.8296	−3.5999	[M+NH_4_]^+^	5.93 × 10^−13^	1.90 × 10^−13^	5.1153
C_53_H_98_O_6_	PPL	848.7693	−1.0460	[M+NH_4_]^+^	2.42 × 10^−4^	1.97 × 10^−5^	4.6773
C_53_H_102_O_6_	PPS	852.7987	−3.3696	[M+NH_4_]^+^	1.39 × 10^−11^	3.15 × 10^−12^	2.4101

P, palmitic; S, stearic; O, oleic; L, linoleic; Ln, linolenic.

**Table 3:** Bioactive compounds (mg/kg) of camellia oils.

	Cold-Pressed	Roast-Pressed	Fresh-Pressed	Refined
α-Tocopherol	96.95 ± 5.32 ^b^	44.22 ± 1.14 ^c^	143.15 ± 3.60 ^a^	3.6 ± 0.46 ^d^
δ-Tocopherol	ND	ND	ND	ND
γ-Tocopherol	ND	ND	ND	ND
Squalene	109.47 ± 3.46 ^a^	87.41 ± 2.07 ^c^	102.08 ± 5.75 ^b^	26.33 ± 1.21 ^d^
β-Sitosterol	92.79 ± 2.44 ^b^	33.2 ± 1.26 ^b^	93.20 ± 2.36 ^a^	ND
Total Polyphenols	19.15 ± 0.5 ^c^	20.73 ± 0.46 ^b^	35.38 ± 0.95 ^a^	ND

The superscript letters indicate the statistical difference in rows at a significance level of 5%.

**Table 4:** Correlations between bioactive compounds, oxidative stability, and antioxidant activity.

	OSI	DPPH Polar Extract	FRAP	ABTS	DPPH Non-Polar Extract	DPPH Whole Oil
OSI	1	0.758 **	0.835 **	0.681 *	0.853 **	0.941 **
α-Tocopherol	0.871 **	0.977 **	0.972 **	0.821 **	0.754 **	0.907 **
Squalene	0.920 **	0.713 **	0.716 **	0.441	0.983 **	0.983 **
β-Sitosterol	0.825 **	0.912 **	0.864 **	0.611 *	0.874 **	0.948 **
Polyphenols	0.977 **	0.830 **	0.910 **	0.812 **	0.755 **	0.898 **

** Correlation is significant at the 0.01 level (2-tailed). * Correlation is significant at the 0.05 level (2-tailed).

## Data Availability

The datasets used and/or analyzed during the current study are available from the corresponding author on reasonable request.

## Supplementary Materials

The following supporting information can be downloaded at: https://www.mdpi.com/article/10.3390/foods11213489/s1, Figure S1: The simplified process flow sheet of four processing methods: cold-pressed, roast-pressed, fresh-pressed and refined; Table S1: Fatty acid compositions of the camellia oils (%); Table S2: Volatile compounds of the camellia oils (mg/kg).
