# Supplementary material for: Typical Characterization of Commercial Camellia Oil Products Using Different Processing Techniques: Triacylglycerol Profile, Bioactive Compounds, Oxidative Stability, Antioxidant Activity and Volatile Compounds
Source: Foods. 2022 Nov 3;11(21):3489. doi: 10.3390/foods11213489 (PMC9658760; doi:10.3390/foods11213489)
Supplement: Supplementary file 1 [file foods-11-03489-s001.zip › foods-1989976-supplementary.pdf]

Typical characterization of commercial camellia oil products using different processing techniques: triacylglycerol profile, bioactive compounds, oxidative stability, antioxidant activity and volatile compounds

supplementary material

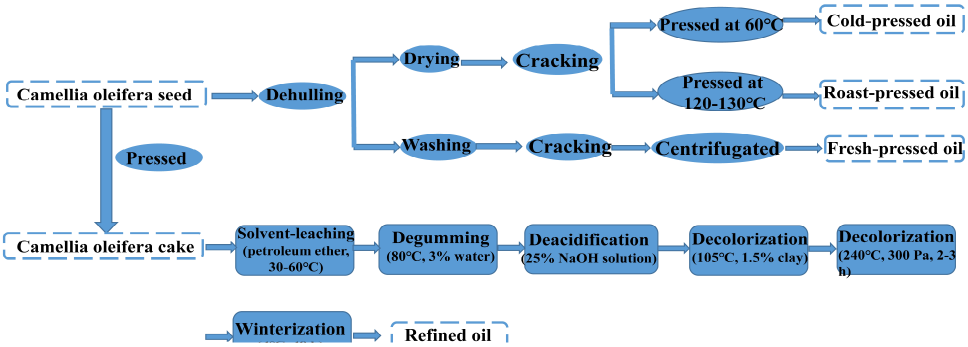

Figure S1. The simplified process flow sheet of four processing methods: cold-pressed, roast-pressed, fresh-pressed and refined.

Table S1. Fatty acid compositions of the camellia oils (%).

Analysts	Cold-pressed	Roast-pressed	Refined	Fresh-pressed
C14:0	0.02 ± 0.00 [a]	0.04 ± 0.00[b]	0.03 ± 0.01 [a]	0.01 ± 0.00 [b]
C16:0	8.45 ± 0.15 [a]	8.21 ± 0.14 [a]	8.43 ± 0.17 [a]	8.27 ± 0.20 [a]
C16:1	0.09 ± 0.01[ab]	0.10 ± 0.01 [a]	0.12 ± 0.02 [a]	0.11 ± 0.01[ab]
C17:0	0.08 ± 0.01 [c]	0.05 ± 0.01 [ab]	0.08 ± 0.00 [ab]	0.07 ± 0.01 [c]
C17:1	0.08 ± 0.01 [a]	0.12 ± 0.02 [bc]	0.10 ± 0.01 [ab]	0.06 ± 0.01 [c]
C18:0	2.05 ± 0.05 [ab]	2.19 ± 0.09 [b]	2.22 ± 0.03 [a]	2.26 ± 0.07 [a]
C18:1	79.67 ± 0.18 [a]	79.27 ± 0.49 [a]	79.29 ± 0.24 [a]	79.12 ± 0.35 [a]
C18:1T	0.03 ± 0.00 [b]	0.06 ± 0.00 [c]	0.08 ± 0.01 [a]	0.02 ± 0.00 [a]
C18:2	8.45 ± 0.14 [a]	8.76 ± 0.19 [a]	8.59 ± 0.25 [a]	8.72 ± 0.19 [a]
C18:2T	0.05 ± 0.01 [a]	0.04 ± 0.00 [a]	0.05 ± 0.00[a]	0.02 ± 0.01 [a]
C18:3	0.55 ± 0.08 [a]	0.59 ± 0.07 [a]	0.46 ± 0.07 [a]	0.48 ± 0.02 [a]
C18:3T	0.02 ± 0.01 [b]	0.01 ± 0.00 [b]	0.04 ± 0.01 [a]	0.02 ± 0.01 [b]
C20:0	0.39 ± 0.03 [a]	0.35 ± 0.03 [a]	0.36 ± 0.04 [a]	0.46 ± 0.06 [a]
C20:1	0.03 ± 0.00 [a]	0.09 ± 0.01 [b]	0.08 ± 0.02 [a]	0.3 ± 0.01 [a]
C22:0	0.04 ± 0.00 [a]	0.12 ± 0.02 [c]	0.07 ± 0.01 [b]	0.08 ± 0.01 [b]
ΣSFA	11.03 ± 0.04 [a]	10.70 ± 0.17 [a]	10.91 ± 0.29 [a]	11.15 ± 0.15 [a]
ΣMUFA	79.87 ± 0.25 [a]	79.84 ± 0.06 [a]	79.87 ± 0.28 [a]	79.59 ± 0.26 [a]
ΣPUFA	9.00 ± 0.24 [a]	9.35 ± 0.2 [a]	9.05 ± 0.12 [a]	9.20 ± 0.18 [a]
ΣTFA	0.10 ± 0.03 [b]	0.11 ± 0.03 [b]	0.17 ± 0.02 [a]	0.06 ± 0.02 [c]

The superscript letters indicate the statistical difference in rows in significant level at 5%.

Table S2. Volatile compounds of the camellia oils (mg/kg).

Analysts	Cold-pressed	Roast-pressed	Fresh-pressed	Refined
2-methyl-2-Butenoic acid ethyl ester	-	-	1.08±0.11	-
Ethyl 2-cyanocrotonate	-	-	0.11±0.01	-
Pentan-2-methylbut-2-enoate	0.15±0.02	-	0.07±0.02	-
Benzoic acid ethyl ester	-	-	2.90±0.16	-
(E)-2-methyl-2-Butenoic acid-3-methylbutyl ester	0.31±0.01	-	-	-
Cyclobutanecarboxylic acid-2-methylbutyl ester	-	-	0.31±0.04	-
Benzeneacetic acid, ethyl ester	-	-	0.42±0.01	-
2-acetyl-4-Pentenoic acid ethyl ester	-	-	-	0.77±0.02
γ-Dodecalactone	-	1.62±0.2	-	-
Benzenepropanoic acid ethyl ester	-	-	0.21±0.02	-
3-phenyl-2-Propenoic acid methyl ester	-	0.96±0.15	0.14±0.02	-
(E)-3-phenyl-2-Propenoic acid ethyl ester	-	-	1.00±0.06	-
Ethyl N-(o-anisyl)formimidate	-	0.52±0.02	-	-
Ethyl p-acetamidobenzoate	-	0.37±0.01	-	-
Ethylene glycol-Adipate-Diethylene glycol	-	-	0.31±0.03	-
Tributyrin	-	-	0.14±0.04	-
Hexadecanoic acid methyl ester	0.20±0.07	0.28±0.04	0.17±0.01	-
Dibutyl phthalate	-	-	0.62±0.04	-
Hexadecanoic acid ethyl ester	0.09±0.01	-	0.64±0.1	-
(Z)-6-Octadecenoic acid methyl ester	0.12±0.01	-	-	-
(E, E)-9,12-Octadecadienoic acid, propyl ester	0.65±0.08	-	0.4±0.02	-
(E)-9-Octadecenoic acid ethyl ester	1.78±0.18	-	1.19±0.15	-
(Z)-9-Octadecenoic acid methyl ester	-	0.27±0.07	0.23±0.06	-
4-Cyclopentene-1,3-dione	-	0.63±0.1	-	-
Acetophenone	-	-	0.35±0.04	-
3-butyl-1,2,4-Cyclopentanetrione	-	0.47±0.03	-	-
Styrene	1.71±0.02	-	-	-
1,2,4-trimethyl-Benzene	-	-	0.26±0.03	-
8-methyl-1-Undecene	0.40±0.03	-	-	-
Hexanoic acid	0.72±0.09	-	-	-
Heptanoic acid	1.79±0.09	-	-	-
Benzoic acid	-	1.14±0.10	-	-
Octanoic acid	10.08±0.26	10.95±0.35	4.30±0.20	-
3-Methyl-2-furoic acid	-	0.38±0.06	-	-
Nonanoic acid	9.79±0.9	10.18±0.32	4.13±0.37	0.10±0.02
3-Furanacetic acid	-	0.65±0.05	-	-
2,6-Dihydroxybenzoic acid	-	-	0.07±0.01	-
Furfural	-	62.61±0.73	-	-
Benzaldehyde	2.16±0.22	-	5.42±0.12	-

(E, E)-2,4-Heptadienal	2.33±0.29	-	-	-
5-methyl-2-Furancarboxaldehyde	-	13.32±0.11	-	-
Octanal	0.68±0.06	0.47±0.02	0.17±0.14	-
1H-Pyrrole-2-carboxaldehyde	-	1.58±0.10	-	-
(E)-2-Octenal	0.32±0.01	-	-	-
Benzeneacetaldehyde	-	3.75±0.22	0.36±0.01	-
Nonanal	6.43±0.17	11.75±0.27	7.86±0.16	0.13±0.03
(E)- 2-Nonenal	0.88±0.05	-	0.94±0.02	-
1-methyl-1H-Pyrrole-2-carboxaldehyde	-	2.19±0.39	-	-
Dodecanal	0.50±0.01	-	-	-
(E, E)-2,4-Nonadienal	1.31±0.02	-	-	-
(E)-2-Decenal	7.55±0.44	13.40±0.26	9.09±0.22	0.32±0.04
(E, Z)-2,4-Decadienal	2.59±0.08	-	-	0.75±0.05
(E, E)-2,4-Decadienal	-	2.15±0.03	1.50±0.04	-
5-Acetoxymethyl-2-furaldehyde	-	0.96±0.06	-	-
2,4-Decadienal	10.03±0.35	6.46±0.07	2.93±0.15	-
2-Undecenal	11.47±0.49	14.4±0.45	9.45±0.12	0.33±0.05
1-(2-furanyl)-Ethanone	-	2.28±0.11	-	-
Eugenol	-	0.24±0.03	-	-
(E)-2-Dodecen-1-ol trifluoroacetate	-	1.47±0.03	-	-
5-butyl-2,3-dimethyl-Pyrazine	-	-	0.38±0.02	-
1-(1H-pyrrol-2-yl)-Ethanone	-	10.59±0.52	-	-
Anethole	0.95±0.07	-	-	-
Cirsiumaldehyde	-	-	2.16±0.18	-
3,4-dihydro-8-hydroxy-3-methyl-1H-2-Benzopyran-1-one	-	0.52±0.07	0.05±0.01	-
3-Methyl-5-nitrosotropone	-	0.12±0.00	-	-
